# Efficient GBA1 editing via HDR with ssODNs by outcompeting pseudogene-mediated gene conversion upon CRISPR/Cas9 cleavage

**DOI:** 10.3389/fgeed.2025.1581743

**Published:** 2025-04-30

**Authors:** Joseph S. Lagas, Monica F. Sentmanat, Xiaoxia Cui

**Affiliations:** Department of Genetics, Genome Engineering and Stem Cell Center at the McDonnel Genome Institute (GESC@MGI), School of Medicine, Washington University in St. Louis, St. Louis, MO, United States

**Keywords:** gene conversion (GC), nonallelic homologous recombination (NAHR), knock out and knock in, gene editing (CRISPR/Cas9), stem cell engineering, pseudogenes

## Abstract

**Introduction:**

CRISPR/Cas9-edited induced pluripotent stem cells (iPSCs) are valuable research models for mechanistic studies. However, gene conversion between a gene-pseudogene pair that share high sequence identity and form direct repeats in proximity on the same chromosome can interfere with the precision of gene editing. Mutations in the human beta-glucocerebrosidase gene (GBA1) are associated with Gaucher disease, Parkinson’s disease, and Lewy body dementia. During the creation of a GBA1 KO iPSC line, we detected about 70% gene conversion from its pseudogene GBAP1. These events maintained the reading frame and resulted from GBA1-specific cleavage by CRISPR/Cas9, without disrupting the GBA1 gene.

**Method:**

To increase the percentage of alleles with out-of-frame indels for triggering nonsense-mediated decay of the GBA1 mRNA, we supplied the cells with two single-stranded oligodeoxynucleotide (ssODN) donors as homology-directed repair (HDR) templates.

**Results:**

We demonstrate that HDR using the ssODN templates effectively competes with gene conversion and enabled biallelic KO clone isolation, whereas the nonallelic homologous recombination (NAHR)-based deletion rate remained the same.

**Discussion:**

Here, we report a generalizable method to direct cellular DNA repair of double strand breaks at a target gene towards the HDR pathway using exogenous ssODN templates, allowing specific editing of one gene in a gene-pseudogene pair without disturbing the other.

## Introduction

Modeling human disease pathology in preclinical models is an essential step in delineating disease mechanisms and the development of targeted therapies. CRISPR/Cas9 is routinely used to introduce genetic perturbations in induced pluripotent stem cells (iPSCs) to model disease causing variants in a patient’s genetic background. However, in some cases, these gene-editing strategies can be complicated by the existence of non-coding or non-functional segments of DNA that resemble functional genes, called pseudogenes (Cheetham et al., 2020; Hanss et al., 2020). Pseudogenes predominantly arise from retrotransposition of mRNA or gene duplications and often share a high degree of homology with their functional counterparts despite having accumulated mutations (Cheetham et al., 2020). There are >14,000 pseudogenes in the human genome, many with >50% sequence homology to their protein coding counterpart (Torrents et al., 2003; Zhang and Gerstein, 2004).

Homologous recombination between a gene and its pseudogene can occur via synthesis-dependent strand annealing (SDSA) or alternate resolution of double Holliday junctions by, for example, but not limited to, the break-induced replication (BIR) pathway, whereby short (<500 bp) sequence is unidirectionally replaced with pseudogene-specific sequence, without crossover, and not leading to large structural changes (Chen et al., 2007; 2010). Gene conversion tracts created by SDSA in mammalian mitotic cells average less than 100 bp following I-SceI-mediated double strand breaks (LaRocque and Jasin, 2010). However, longer tracts have been observed in other organisms, such as 280 bp in budding yeast and 471 bp in *Drosophila* (Cho et al., 1998; Mansai et al., 2011). Gene conversion typically occurs between sequences with >90% identity, is positively correlated to length of sequence similarity and negatively correlated to distance between homologous templates (Schildkraut et al., 2005; Chen et al., 2007; 2010). Gene conversion leads to sequence diversity, contributing to the evolutionary trajectory of gene families like the human leukocyte antigen (HLA) loci that require high variability for environmental adaptability (Adamek et al., 2015) but also contributes to numerous genetic diseases, such as Gaucher Disease, an autosomal recessive lysosome storage disorder caused by mutations in GBA1 (Chen et al., 2007; Hruska et al., 2008).

Recombination events between GBA1 and its pseudogene, GBAP1, 16 kb downstream on the same chromosome, contribute a spectrum of disease-causing recombinant alleles at GBA1 spanning from intron 2 to exon 11 (Hruska et al., 2008). A study of 240 patients with Gaucher Disease found that 12% of alleles at GBA1 were recombinant alleles, most arising from gene conversion, nonreciprocal recombination that contributes short tracts of unidirectional sequence, or reciprocal recombination producing larger structural variation (e.g., deletion events) through nonallelic homologous recombination (NAHR) (Tayebi et al., 2003). Structural variation arising from NAHR involves a double Holliday junction that leads to crossover between high homology sequences that are nonallelic (e.g., pseudogene and parental copy), resulting a byproduct of deletions for intrachromosomal NAHR or concomitant deletions and duplications for inter-chromosomal NAHR when the recombining regions are in the same orientation (Gu et al., 2008). Recurrent copy number variation mediated by NAHR is often flanked by low-copy repeats and are associated with genetic disorders such as Prader-Willi, DiGeorge, and Charcot-Marie-Toothe disease (Dittwald et al., 2013).

GBA1 has also been implicated in Parkinson’s disease and certain metabolic disorders when mutated. However, the determination of the exact disease-causing variants has been complicated by the existence of the GBA1 pseudogene, GBAP1, located 16 kb downstream in the same orientation as its functional counterpart. GBAP1 shares >96% identity to GBA1, leading to misalignments during genome assembly and transcript quantification by short read sequencing (Woo et al., 2021). Long-read sequencing of transcripts has improved mapping across highly repetitive regions, unveiling pseudogene functional roles, through protein-coding and noncoding transcripts (Troskie et al., 2021; Qian et al., 2022). Recently, GBAP1 was found to generate protein with activity that is independent of the canonical GBA1 lysosomal hydrolase function and transcripts with cell type specificity (Gustavsson et al., 2024). When attempting to edit the GBA1, it is important to avoid perturbing the GBAP1 gene.

Here, we sought to generate a GBA1 KO in an iPSC line by targeting exon 6 using CRISPR/Cas9. We observed that 70% of alleles in the CRISPR RNP transfected pools had no indels, whereas indels are a signature editing outcome via nonhomologous end joining (NHEJ) at double strand breaks (DSBs). Instead, these alleles had single nucleotide variants (SNVs) near the gRNA cut site, largely matching the GBAP1 sequences but not identical, indicative of highly efficient homology-dependent repair of DSBs. We reasoned DNA repair was predominantly via gene conversion, homologous recombination using GBAP1 as template and effectively quenching NHEJ-mediated KO. We sequenced the genomic region spanning GBA1 and GBAP1 in iPS1 using a Nanopore long-read sequencing-based method, LOCK-seq (Sentmanat et al., 2024), and confirmed that SNVs in edited GBA1 alleles match those in GBAP1. We also detected NAHR-mediated deletion between the GBA1 and GBAP1 loci.

To compete with gene conversion, i.e., homologous recombination using the pseudogene as template, we co-transfected Cas9/gRNA RNP with two single-stranded oligodeoxynucleotide (ssODN) donors carrying out-of-frame deletions as HDR templates. This approach resulted in >10% knock-in (KI) efficiency in the pool and a reduced gene conversion rate, ultimately enabling the successful isolation of biallelic out-of-frame clones, whereas the rate of large deletions via NAHR was not impacted. Here, we present evidence that GBAP1 serves as a preferential HDR template for repairing DSBs at GBA1 exon 6 and introduce a novel and generalizable strategy to outcompete gene conversion from highly similar pseudogenes and improve editing efficiency of the target genes.

## Materials and methods

### Cell culture

Human iPSCs used in this study were iPS1, an unpublished line reprogrammed from hepatic fibroblasts from a healthy control, and iPS2, reprogrammed from renal epithelial cells isolated from urine (Chen et al., 2023). Both lines were cultured in mTeSR Plus (Stemcell Technologies, cat#.100-1130) on Matrigel-coated plates (Corning, United States) and passaged using ReLeSR (Stemcell Technologies, cat#.100-0483). HEK293T cells were cultured in Dulbecco’s Modified Eagle’s Medium (ThermoFisher, cat#.11965092) with 10% FBS (ThermoFisher, cat.#A5670701) and passaged using 0.25% trypsin (ThermoFisher, cat#.25200056). All cell lines were maintained in tissue culture incubators under conditions of 37°C, 95% air, and 5% CO_2_ in a humidified incubator. All cultures were routinely tested for the absence of *mycoplasma* and authenticated by STR profiling. iPSCs were confirmed to have normal karyotype using G banding.

### gRNA and donor ssODN design

gRNAs were designed using an in-house CRISPR design algorithm which combines specificity scores from the Zhang lab (Hsu et al., 2013), activity prediction scores by the Doench lab (Doench et al., 2016), as well as SNP check using dbSNP. Specificity scores are the primary factor considered in gRNA selection. The spacer sequence for the gRNA used to target exon 6 was 5′-CCA​TTG​GTC​TTG​AGC​CAA​GT -3′, and the gRNA was purchased as a one-piece synthetic molecule, Alt-R CRISPR-Cas9 sgRNA (IDT, Coralville, IA), with standard modifications. The ssODNs were designed with 60 bases of homology arms to the target site upstream and downstream of the gRNA cut site with two phosphorothioate (PTO) bonds to protect from exonuclease activity at each of the terminal 5′ and 3′ ends of the molecules. The sequence for the 7 bp deletion including PTO modifications denoted by asterisks 5′-c*c*ctg​cag​ttg​gcc​cag​cgt​ccc​gtt​tca​ctc​ctt​gcc​agc​ccc​tgg​aca​tca​ccc​act​aga​cca​atg​gag​cgg​tga​atg​gga​agg​ggt​cac​tca​agg​gac​agc​ccg​gag​aca​tct​acc​ac*c*a-3′ and 10 bp deletion 5′-c*c*ctg​cag​ttg​gcc​cag​cgt​ccc​gtt​tca​ctc​ctt​gcc​agc​ccc​tgg​aca​tca​ccc​act​cca​atg​gag​cgg​tga​atg​gga​agg​ggt​cac​tca​agg​gac​agc​ccg​gag​aca​tct​acc​ac*c*a-3′, both obtained as Ultramers from IDT.

### Nucleofections

Nucleofections were performed using the Lonza Bioscience 4D-Nucleofector P3 kit per the manufacturer’s instructions. Briefly, iPSCs were single-cell dissociated with StemPro Accutase (ThermoFisher, cat.#A1110501) to collect one million cells per reaction. Cells were pelleted at 300 × g and washed once with PBS (ThermoFisher, cat.#10010023). To complex gRNA with Cas9 protein, 2 µL IDT Cas9 protein (10 ug/uL, IDT, cat.#1081058) was combined with 2 µL of gRNA (100 µM) and incubated at room temperature for 10–30 min. Before adding cells to the Cas9-gRNA RNP, 1 µL of ssODN (100 µM) was added to the reaction. Cells were resuspended in 100 µL P3 nucleofection solution and added to the RNP reaction. The cell suspension was transferred to a large cuvette and nucleofected using the CA137 program. Cells were replated in Matrigel-coated plates in mTeSR Plus with 10 µM ROCK inhibitor (MilliporeSigma, cat.#SCM075). ROCK inhibitor was removed after 24 h and the cells were cultured for an additional 2 days before harvesting for NGS.

### Next-generation sequencing and analysis

Nucleofected pools of iPSCs were lysed in QuickExtract Solution (Biosearch Technologies, cat#.QE09050) following the manufacturer’s instructions. The target region is then amplified by PCR in two steps using universal tails appended to genomic-specific primers. The universal tail sequences are: 5′ – CAC​TCT​TTC​CCT​ACA​CGA​CGC​TCT​TCC​GAT​CT – 3′ for the forward primer and 5′ – GTG​ACT​GGA​GTT​CAG​ACG​TGT​GCT​CTT​CCG​ATC​T – 3′ for the reverse primer. All primers used in this study are listed in Supplementary Table S4. These tails allow for unique indexes and Illumina P5/P7 adapter sequences to be added for the second round of PCR. The tails were attached to the genomic-specific primer sequences: 5′ – CCT​GAT​GTC​TGG​GGG​TTG​AG – 3′ forward (full sequence including tail is 5′-CAC​TCT​TTC​CCT​ACA​CGA​CGC​TCT​TCC​GAT​CTCCT​GAT​GTC​TGG​GGG​TTG​AG-3′) and 5′ – ACA​GAT​CAG​CAT​GGC​TAA​ATG​G – 3′ reverse (full sequence including tail is 5′- GTG​ACT​GGA​GTT​CAG​ACG​TGT​GCT​CTT​CCG​ATC​TACA​GAT​CAG​CAT​GGC​TAA​ATG​G -3′). Indexing of step 1 product was performed using 0.1X volume from step 1 with indexing primers (list of indexing primers listed in Supplementary Table S4). Products generated from step 2 PCRs were submitted to the sequencing lab at the Center for Genome Sciences and Systems Biology affiliated with Washington University in St. Louis for a 2x250 run on the MiSeq. PCRs were performed using REDTaq ReadyMix PCR Reaction Mix (MilliporeSigma, cat.#R2523-100RXN) according to the manufacturer’s instructions. NGS data was analyzed using an in-house script (Connelly and Pruett-Miller, 2019). Briefly, the script uses FASTQ files from amplicon-sequencing data as input and searches for short (<30 bp) user-provided sequence proximal and/or overlapping with the primer-binding sites to query reads for the presence of a list of wild-type or knock-in sequence (Supplementary Table S5 lists full data output). The output also parses the most frequently occurring reads for the region of interest, allowing for quantification of percent of total for each population.

### LOCK-seq

LOCK-seq was performed as described in Sentmanat et al. (2024). Briefly, genomic DNA was isolated from tissue and cell pellets using the Monarch Genomic DNA Purification Kit (NEB, cat.#T3010S), according to the manufacturer’s instructions. Fragmentation was performed using 500 ng input with the seqWell LongPlex Long Fragmentation kit (seqWell, Beverly, MA). Custom capture probes (JSL001.GBA1-1-10, Supplementary Table S4) were from xGen Custom Hybridization Probe Panels (IDT, Coralville, IA). In-solution hybridization reactions were performed for 16–18 h s at 65°C using the xGen Hybridization and Wash Kit (IDT, cat.#1080577) with Universal Blocker NXT (IDT, cat.#1079584), according to the manufacturer’s protocol. Ligation of Oxford Nanopore sequencing adapters was performed using the Ligation Sequencing Kit V14 (Oxford Nanopore Technologies, cat.#SQK_LSK114) and loaded onto an Oxford Nanopore Technologies flow cell (FLO-MIN114).

### Bioinformatics analysis

Base calling was performed using Dorado (v0.7), and samples were demultiplexed with an in-house python script. Briefly, no trimming was performed, and for the in-house demultiplexing script, exact index matches for both indexes were required. Reads with >1 match for either index or lacking one index were excluded. Capture efficiency was determined using flagstat output (total reads mapped to transgene/total reads) and length of mapped reads calculated using FASTQ files derived from transgene mapped BAM files for each sample. FASTQ files were aligned to the target region (GBAP1-GBA1 at chr1:155,213,240-155,240,028 of the hg38 reference sequence) using Minimap2 (v2.28) and Samtools (v1.20). BAM files were used to build consensus sequences using Canu (v2.2) and visualized using IGV.

## Results

### CRISPR/Cas9-mediated targeting of GBA1 at exon 6 predominantly results in gene conversion from GBAP1

**FIGURE 1:**
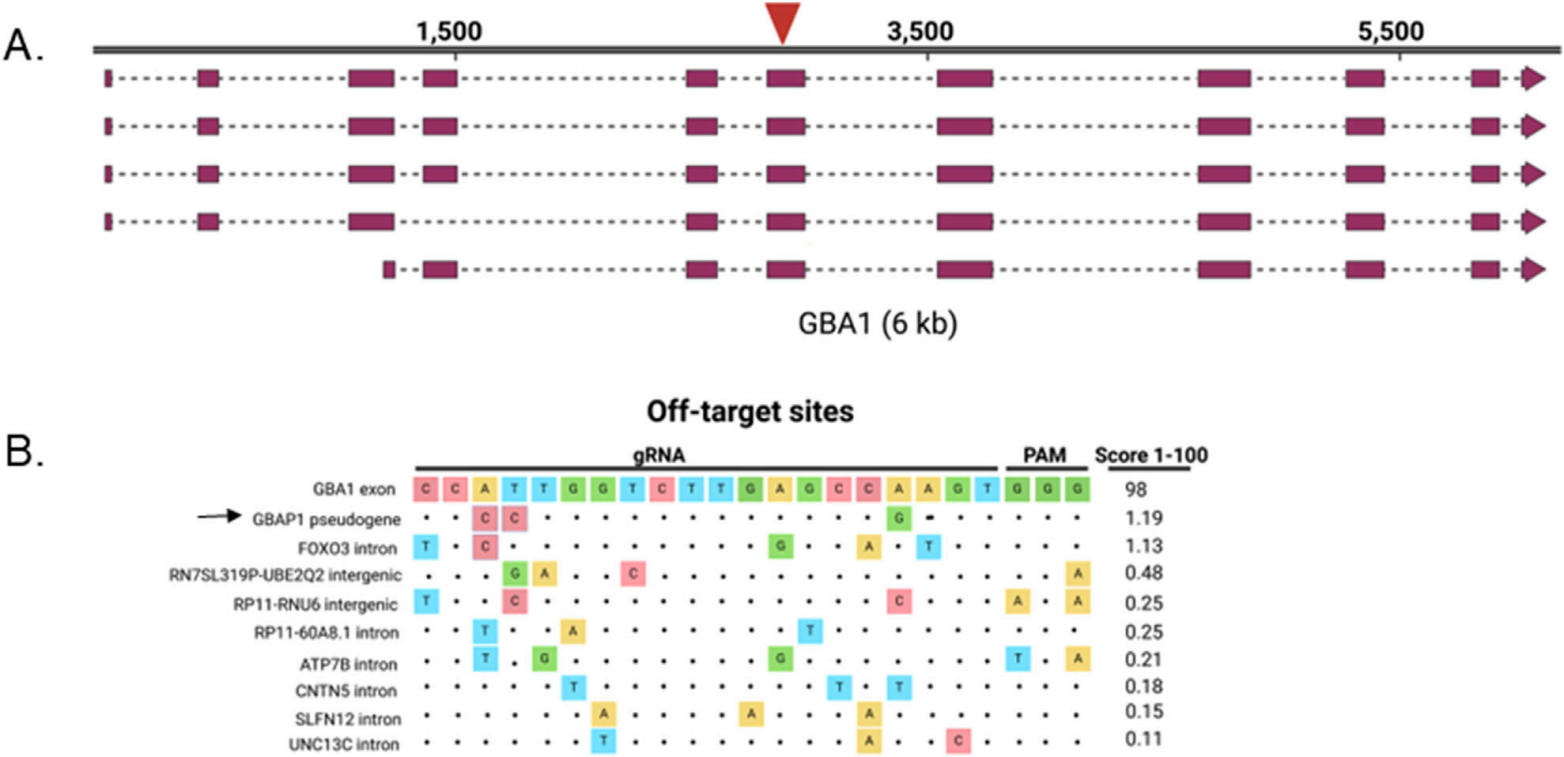
GBA1 target locus. **(A)** GBA1 transcripts and gRNA target site (red arrow). **(B)** Alignment of top 10 off-target sites ordered from highest off-target score (top) to lowest for the gRNA used to target GBA1 exon 6. Score is the MIT specificity score (Hsu et al., 2013) with scores of ≤ 50 being poorly specific. Arrow points to GBAP1 off-target site. Dots indicate sequence homology.

**FIGURE 2:**
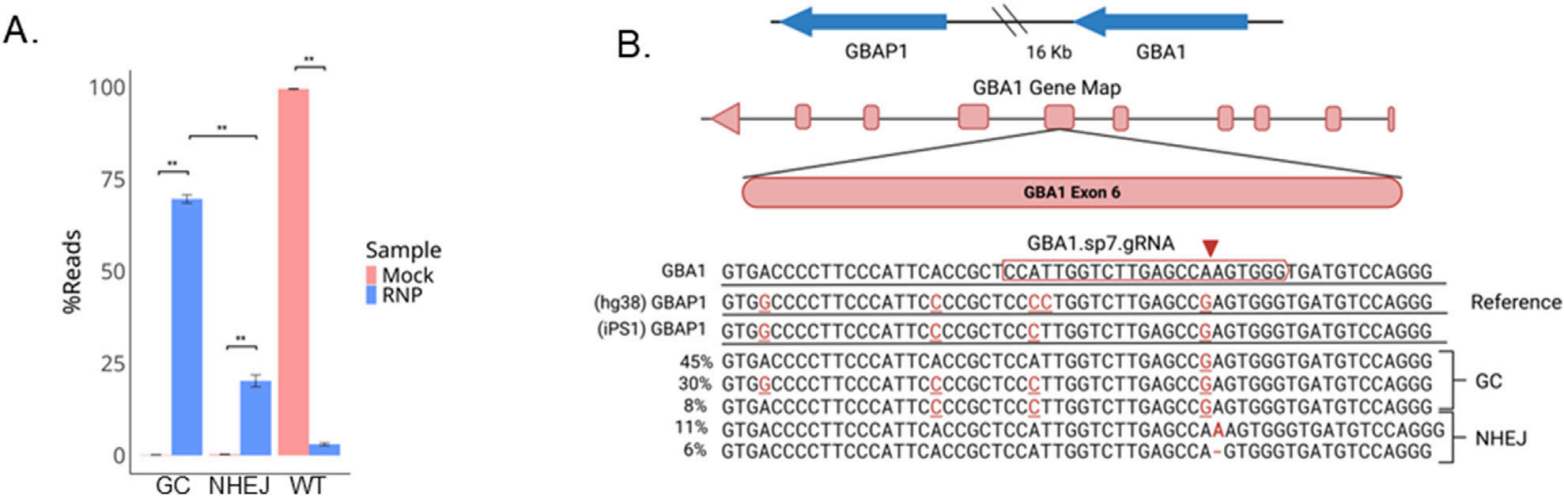
Gene conversion is the predominant outcome after CRISPR/Cas9 editing at GBA1. **(A)** Barplot of percentage of reads with gene conversion (GC, no indels), wild-type, or NHEJ for the ± RNP edited pool and **(B)** alignments of the five most frequently occurring reads. **P < 0.001 by Student’s t-test. The data shown represents the mean ± SD (n = 3).

To disrupt the GBA1 gene, we chose a gRNA that targets a common exon to all isoforms, exon 6, with high specificity (Figures 1A, B) (Doench et al., 2016; Haeussler et al., 2016). iPS cells (line iPS1) were nucleofected with CRISPR/Cas9 RNP and sampled 72 h later. Amplicon sequencing of the transfected pool revealed that despite less than 2% of wild-type (WT) sequence remaining at the target site, instead of indels, greater than 70% of the reads had single nucleotide variants that are present in its pseudogene, GBAP1, indicating DNA repair via gene conversion (GC) (Figure 2A). NHEJ is the predominant repair pathway for CRISPR-mediated double strand breaks in the cell, by which simple KOs are commonly achieved. Alignments revealed that most of the base changes in the reads were proximal to the CRISPR/Cas9 cut site, largely matching the GBAP1 reference sequences, yet not identical (Figure 2B). The mock transfected parental cells produced only wild type reads, supporting that the base changes were not a result of template switching between GBA1 and GBAP1 loci during PCR. The CRISPR-dependent base changes suggest that gene conversion with GBAP1 is potentially the predominant repair pathway used at this site, not the typical NHEJ that dominates CRISPR/Cas9 edited sites.

**FIGURE 3:**
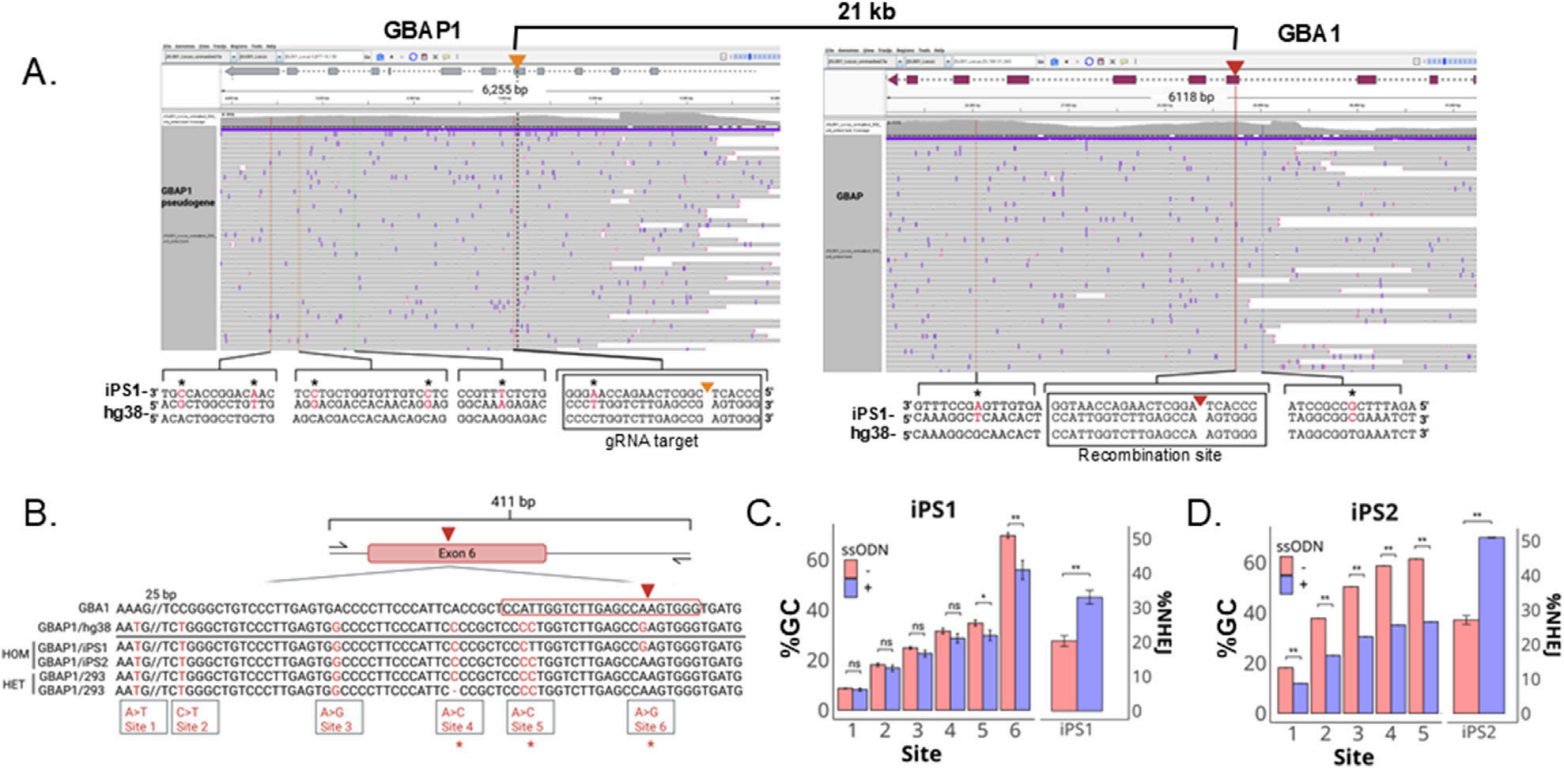
Decreased gene conversion (GC) rate with the addition of ssODNs. **(A)** The parental iPS1 line was sequenced using LOCK-seq. Shown are the raw reads for parental iPS1 as screenshots of the IGV alignments at GBA1 and GBAP1 for iPS1 with hg38 as reference, to highlight the differences present in the iPSC parental genotype. Asterisks indicate variants present in the iPSC line but not in the hg38 reference. **(B)** Amplicon highlighting six sites that differentiate GBA1 and GBAP1 (red bases) in iPSC lines iPS1 and iPS2 as well as HEK293T cells with asterisks to highlight variants that differ across the lines and **(C)** percent gene conversion (GC) and NHEJ across CRISPR RNP transfected iPS1 and **(D)** iPS2 pools ± ssODN. **P < 0.001, *P < 0.05, ns is P > 0.05 by Student’s t-test. The data shown represents the mean ± SD (n = 3).

GBAP1, a GBA1 pseudogene 16 kb downstream of GBA1, shares 96% sequence identity to GBA1 and interferes with GBA1 genotyping (Woo et al., 2021; Orimo et al., 2024). To confirm that the observed SNPs in the edited pool were products of recombination with GBAP1, we first performed LOCK-seq, a target captured long-read sequencing with probes spanning the GBA1 and GBAP1 genomic region in the parental iPS1 line (Sentmanat et al., 2024). The homologous sequence to the chosen gRNA target site in GBAP1 is 21 kb from the target site in GBA1, and the iPSC line has a C>T point mutation in the GBAP1 gene, compared to the hg38 reference genome, that was also in the edited pool (Figure 3A), explaining the discrepancy we observed in Figure 2B. In total, eight SNPs were identified between GBA1 and GBAP1 in the parental iPSC line, all with allele frequencies consistent with homozygosity (≥90%, Supplementary Table S1). The primers used for amplicon sequencing of edited cells were designed to specifically amplify the GBA1 locus, generating a 411 bp amplicon for Illumina sequencing. The amplicon covers six polymorphisms that distinguish GBA1 and GBAP1: four upstream of the gRNA target site and two within the gRNA target sequence, the latter accountable for the gRNA specificity towards GBA1 (Figure 3B).

NHEJ is usually more efficient than HDR (Certo et al., 2011; Chu et al., 2015; Chien et al., 2020). However, gene conversion is effectively outcompeting NHEJ for the GBA1 gene. We reasoned that many copies of single-stranded oligodeoxynucleotide (ssODN) donors could potentially compete and reduce the gene conversion rate, enabling more efficient KO. We designed two ssODNs, one with a −7 bp deletion and a second with a −10 bp deletion, flanked by 60 base homology arms. DNA repair through HDR with either donor will result in premature stop codons within exon 6, triggering nonsense-mediated decay of the GBA1 mRNA. In the pools transfected with CRISPR RNP alone, we observed an increasing percentage of GBAP1-specific polymorphisms approaching the gRNA cut site, with gene conversion detected as far as >50 bp upstream from the cut site (Figure 3C). Co-transfection of both ssODNs with CRISPR RNP decreased gene conversion by as much as 15% with a concomitant increase in percentage of alleles with 7 and 10 bp deletions. This data suggests that gene conversion is more efficient than NHEJ at GBA1, likely due to the proximity and length of homology between the sites. The addition of ssODNs for the desired modifications can aid in the suppression of the undesired gene conversion repair outcome.

To determine whether using ssODNs to outcompete gene conversion is generally applicable to other cell lines, including non-stem cell lines, we tested a second iPSC line (iPS2) and HEK293T cells using the same gRNA and ssODNs. iPS2 showed the same trend as iPS1 (Figure 3D). Even though the SNP at site 6 is absent in the GBAP1 gene in iPS2, the gRNA was specific and only cleaved GBA1. The GBAP1 site was intact in RNP transfected iPS2 cells (Supplementary Table S2). In both iPSC lines, the closer the SNP is to the cut site, the higher the conversion rate, indicating that the predominant repair pathway was in fact via HDR using GBAP1 as donor template, instead of deletion between the homologous repeats via nonallelic homologous recombination. Interestingly, in iPS1 we observed a drastic drop of conversion rate between sites 5 and 6, implying the repair process is most efficient within this 12 bp window. iPS2 has no mismatch at site 6 between the pair of loci and cannot be assessed similarly.

**FIGURE 4:**
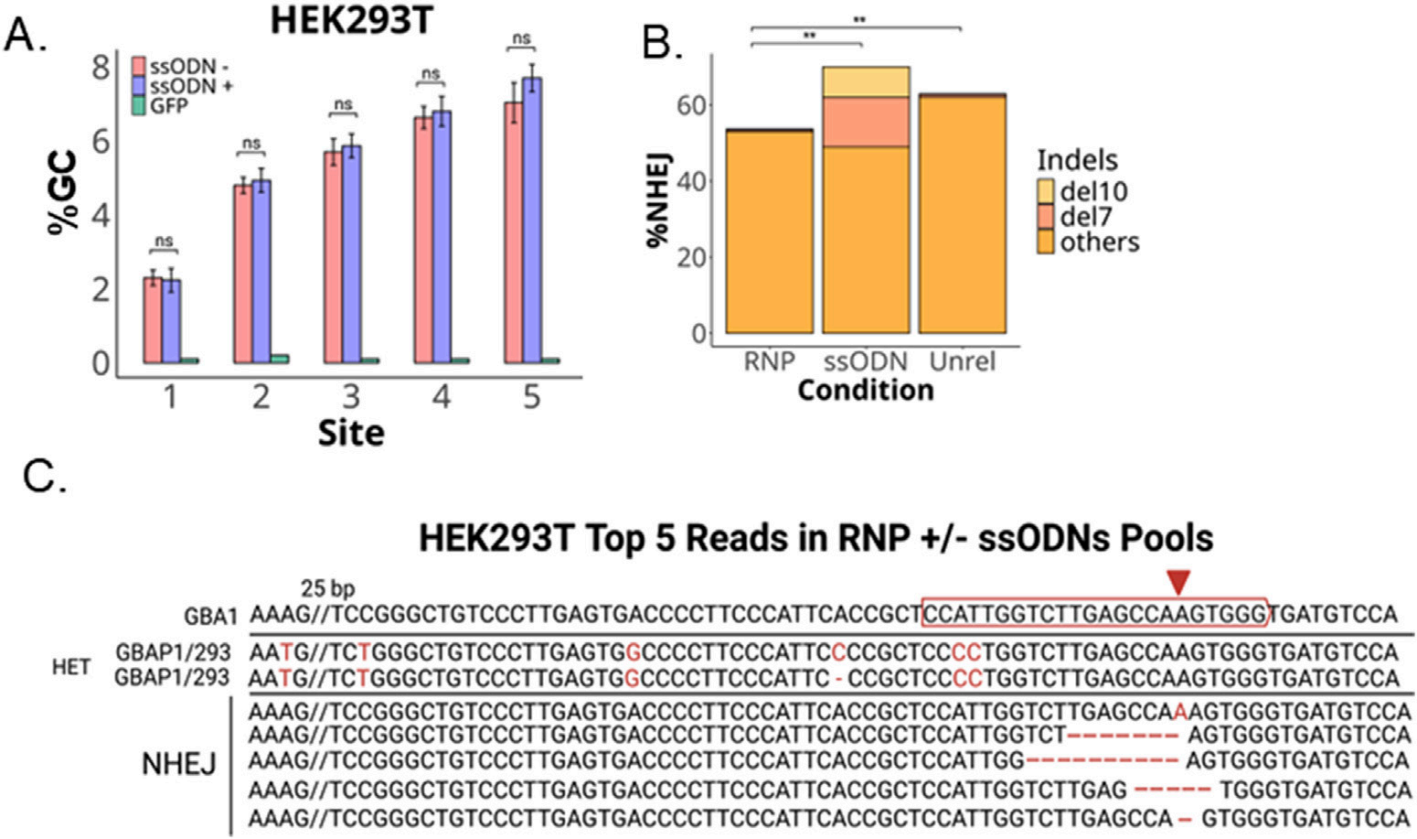
GC and NHEJ in HEK293T. **(A)** Bar plot of percent GC across sites expected to undergo recombination with GBAP1. **(B)** Total percent NHEJ ± ssODNs with −10 bp and −7 bp indels or unrelated ssODN without homolgy to GBA1 locus with significance in total %NHEJ **(C)** Alignment of top five reads in transfected pools. **P < 0.001 and ns is P > 0.05 by Student’s t-test. The data shown represents the mean ± SD (n = 3).

The HEK293T cells also lack the mismatch at site 6 and are heterozygous for a 1 bp deletion at Site 4 (Figure 3B). Compared to the iPSCs, the pool of HEK293T cells transfected with CRISPR RNP had a much higher NHEJ rate (over 50%) and a much lower percentage of gene conversion, 7% (vs. over 70% in iPSCs) (Figure 4A). We did not observe a reduction in conversion alleles when ssODNs were included despite 20% HDR-mediated −7 and −10 alleles (Figure 4B). Alignment of the top five reads in CRISPR RNP ± ssONDs supports that indel-containing reads lack nearby GBAP1 variants (Figure 4C).

**FIGURE 5:**
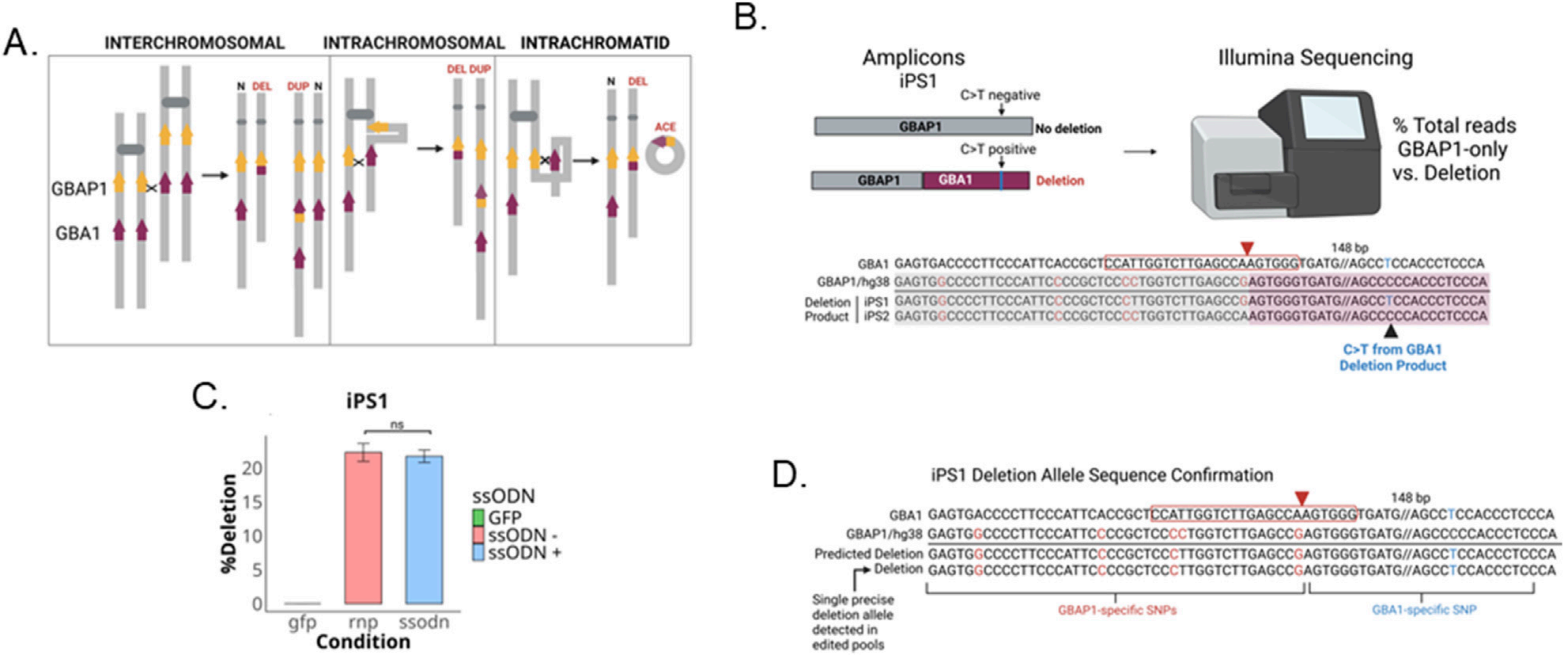
Homology-directed gene fusion between GBAP1 and GBA1. **(A)** Schematic of NAHR outcomes (adapted from Gu et al., 2008). **(B)** Schematic of gene fusion product generated from deletion between loci. **(C)** Barplot of percent of total reads positive for GBA1-specific C>T variant indicative of deletion product **(D)** Alignment of perfect deletion product detected in CRISPR RNP ± ssODN pools of iPS1. ns is P > 0.05 by Student’s t-test. The data shown represents the mean ± SD (n = 3).

The resolution of inter- or intra-chromosomal and intra-chromatid NAHR results in deletions when the two loci are in the same orientation on a chromosome arm (Figure 5A). To assess whether transfected iPSC pools are undergoing a homology-directed deletion event that results in a fusion of the GBAP1 and GBA1 loci with deletion of the intervening genomic region, we performed NGS on PCR amplicons targeting the deletion junction. The primers were designed to simultaneously amplify both the deletion product as well as the GBAP1 locus, producing amplicons of the same size but differ at a single T/C SNV (chr1:155,238,392, hg38) (Figure 5B). This allowed us to quantify the percentage of reads of the deletion product and unmodified GBAP1 locus, respectively. The deletion reads contain a GBA1-specific variant 148 bp away from cut site (Supplementary Figures S1, S5B). A single deletion product was detected in 20% of total reads in iPS1 pools with and without co-transfection of ssODNs and was absent in the untransfected parental line (Figure 5C). The same deletion allele was present in all replicates transfected with RNPs, representing an abundant repair product besides those via gene conversion (Figure 5D). We further confirmed the presence of deletion products using locus-specific primers that bind to either GBAP1- and GBA1-specific variants present in HEK293T cells (Supplementary Figures S2A, B). iPS2 lacks the unique GBA1-specific variant within the genotyping window suitable for NGS and could not be assessed the same way. Only the perfect deletion junction was present with the expected variants identified in iPS1 and HEK293T cellswith or without co-transfection of ssODNs (Supplementary Figure S2C). Additionally, the HEK293T cells have two deletion alleles, sharing identical junction but containing two heterozygous SNVs, one within GBAP1 and the other, the GBA1-specific SNV 148 bp from the cut site (Supplementary Figures S3A, B). The absence of indels both at the breakpoint of the single deletion product detected and at the GBAP1 locus in RNP transfected pools implies the deletion more likely resulted from NAHR than from NHEJ-mediated fusion following the gRNA cutting of both GBA1 and GBAP1 (see Supplementary Table S2), although we cannot completely exclude the possibility of the latter. NHEJ-mediated deletions are usually accompanied by indels at the deletion junction (Canver et al., 2014).

### Introduction of ssODNs containing out-of-frame indels for GBA1 enabled successful isolation of GBA1 KO clones

**FIGURE 6:**
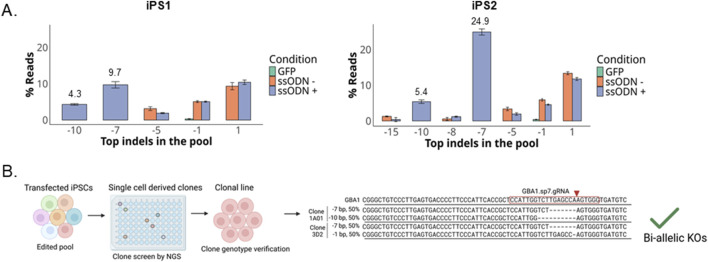
Compound heterozygous knockin clones identified. **(A)** Indel distribution as percent of total reads in two iPSC lines transfected with RNP ± ssODNs, the data shown represent the meant ± SD (n = 2). **(B)** Workflow diagram for clonal line isolation and alignment of compound heterozygous clones with reads mapping to GBA1.

By co-delivery of ssODNs carrying out-of-frame deletions with RNPs, we observed that the indels introduced by the ssODNs were among the most abundant GBA1 alleles in the transfected pools in both iPSCs (Figure 6A). We screened 126 single-cell derived iPS1 clones and identified 22 KO and three wild-type isogenic clones (Figure 6B; Supplementary Table S3). Across clones with alleles from gene conversion, SNV conversion rate correlates to its distance from the cut site. Many clones only possess the GBAP1-specific polymorphisms closest to the cut site for at least one allele, in agreement with the data on the edited pool (Figure 3C). As predicted from the edited pool data, 36% (8/22) of KO clones had one or both donor-specified KO alleles, two of which were biallelic clones containing one of each donor sequence (Supplementary Table S3). Indeed, one advantage of using two donors is that loss-of-heterozygosity at the target site, as reported to occur in up to a third of edited clones, can be avoided by selecting clones with distinct indels (Kosicki et al., 2018; Boutin et al., 2021).

## Discussion

Mutations in GBA1 is one of the most common genetic risk factors for Parkinson’s disease and Gaucher’s disease. Some GBA1 mutations are predicted to be loss-of-function leading to reduced protein levels of beta-glucocerebrosidase, whereas others are suspected of being gain-of-function and require a different therapeutic approach (Smith et al., 2022; Huh et al., 2023). Modeling such mutations in iPSCs is a scalable means to survey the phenotypic outcome across mutations. The generation of GBA1 KO iPSC lines as a disease model was hindered by the existence of GBAP1, a GBA1 pseudogene, which shares 96% identity to the coding sequences of the GBA1 gene. Previous literature has described challenges to CRISPR gene editing of targets with pseudogenes due to limited regions with uniquely targetable sequences and undesired gene conversion or NAHR events (Klatt et al., 2019; Javidi-Parsijani et al., 2020; Wrona et al., 2020; Shaw and Estus, 2021; Yanovsky-Dagan et al., 2022).

Here we showed that double strand breaks at GBA1 by CRISPR/Cas9 were repaired predominantly by base changes instead of indels. Using LOCK-seq, we identified eight polymorphisms across GBA1 and GBAP1 that are unique to this iPSC line, emphasizing the need to carefully sequence the parental line across this highly variable region. The base changes observed match perfectly to sequences of GBAP1, a GBA1 pseudogene 16 kb downstream on the same chromosome. Bases closer to the cut site were converted more frequently than those further away (Figure 3), suggesting high-efficiency gene conversion utilizing GBAP1 as a repair template. In addition, we also detected repair products via the NAHR pathway, which results in a deletion between the direct repeats with the fusion junction exactly at the cut sites.

To generate a GBA1 KO model, we designed two ssODNs containing out-of-frame indels to serve as HDR template and co-nucleofected the cells with CRISPR RNP to outcompete GBAP1-mediated gene conversion. The inclusion of these ssODNs increased the indel percentage in the pool by ∼17% (Figure 3C), which proved to be sufficient to produce multiple GBA1 KO clones (Figure 6B; Supplementary Table S3). We also show that both donors can serve as templates for HDR in the same cell, assisting with the recovery of KOs with distinct alleles that ensure copy number is intact. We outline here a novel strategy for achieving desired CRISPR/Cas9 gene editing complicated by high frequency pseudogene-mediated gene conversion events.

The interference we observed is unlikely to be limited to GBA1 and GBAP1 genes but potentially applicable to other gene-pseudogene pairs that share high sequence identity and are in proximity to each other on the same chromosome. Thus far, no methods have been reported for preventing or lowering the efficiency of pseudogene-mediated conversion events to ensure only the target gene is edited as desired. To generate a GBA1 KO *in vitro* iPSC model we utilized a novel approach involving the use of ssODNs to outcompete the GBAP1 as HDR template.

The approach described herein is technically simple and cost-effective. ssODNs carrying the desired mutations flanked by two 60-base homology arms can be designed and ordered from various vendors quickly and included in nucleofection reactions along with Cas9-RNP. The inclusion of ssODNs reduced the pseudogene-mediated gene conversion events by ∼10% at the GBA1 locus, allowing ready isolation of single-cell clones with two out-of-frame alleles.

To address if the gene conversion observed was generalizable to other cell lines, we next tested competition between ssODN-mediated HDR and gene conversion in a second iPSC line and HEK293T cells, an SV40 transformed clone of the human embryonic kidney 293 cell line. The second iPSC line produced similar results as in iPS1 line, however, we did not observe significant gene conversion rate in HEK293T cells. Further studies are needed to demonstrate whether this is common in transformed cells or specific for the GBA1/GBAP1 pair in HEK293T cells.

Our report highlights the complexity of targeting genes with pseudogene counterparts for CRISPR-Cas9 gene editing. DSBs in GBA1 by CRISPR-Cas9 resulted in a high frequency of GBAP1 pseudogene-mediated gene conversion events that can be circumvented by the inclusion of ssODNs specifying the mutation of interest, out-of-frame indels in this case, as competing templates, for the successful creation of GBA1 KO. Here we have shown that the novel approach of utilizing ssODN donors is a simple and straightforward method to overcome the interference of pseudogene-mediated gene conversion events during CRISPR-Cas9 gene editing and that LOCK-seq is a useful targeted sequencing method to verify precise editing at the gene of interest even in the presence of highly homologous pseudogene nearby.

## Data Availability

The data presented in this study are included in the article/Supplementary Materials. Further inquiries can be directed to the corresponding author.
