# Supplementary figures and images for: Efficient GBA1 editing via HDR with ssODNs by outcompeting pseudogene-mediated gene conversion upon CRISPR/Cas9 cleavage
Source: Front Genome Ed. 2025 Apr 30;7:1581743. doi: 10.3389/fgeed.2025.1581743 (PMC12075325; doi:10.3389/fgeed.2025.1581743)

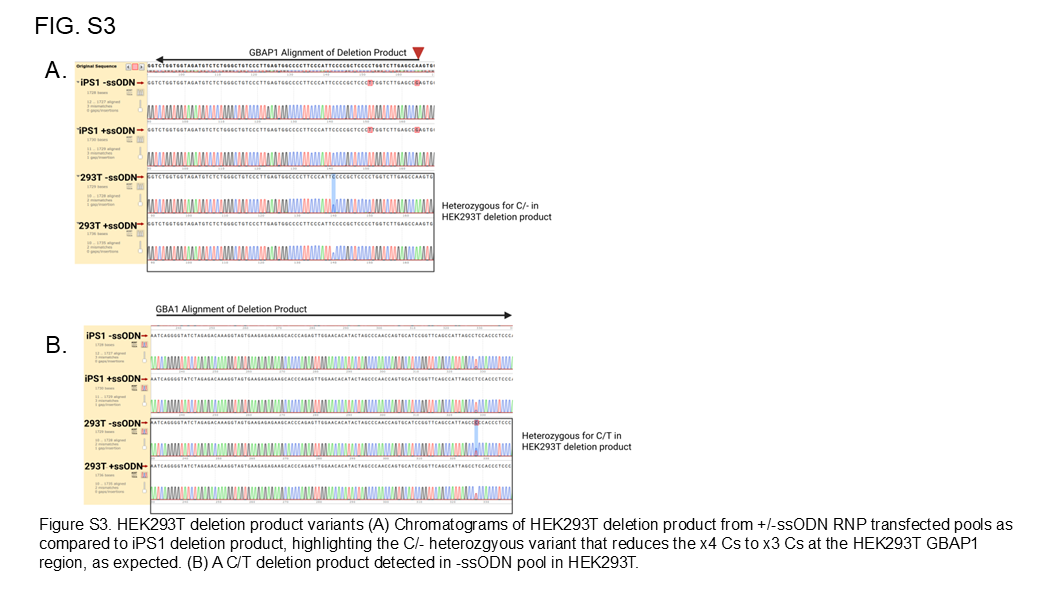

Supplement: Supplementary file 3 [file Image3.tif]

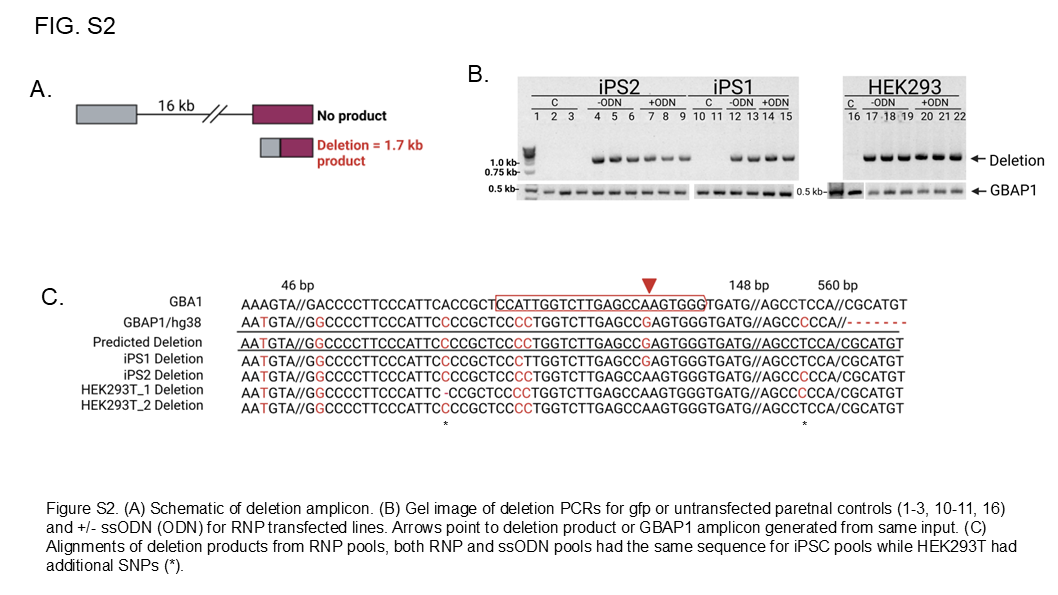

Supplement: Supplementary file 4 [file Image2.tif]

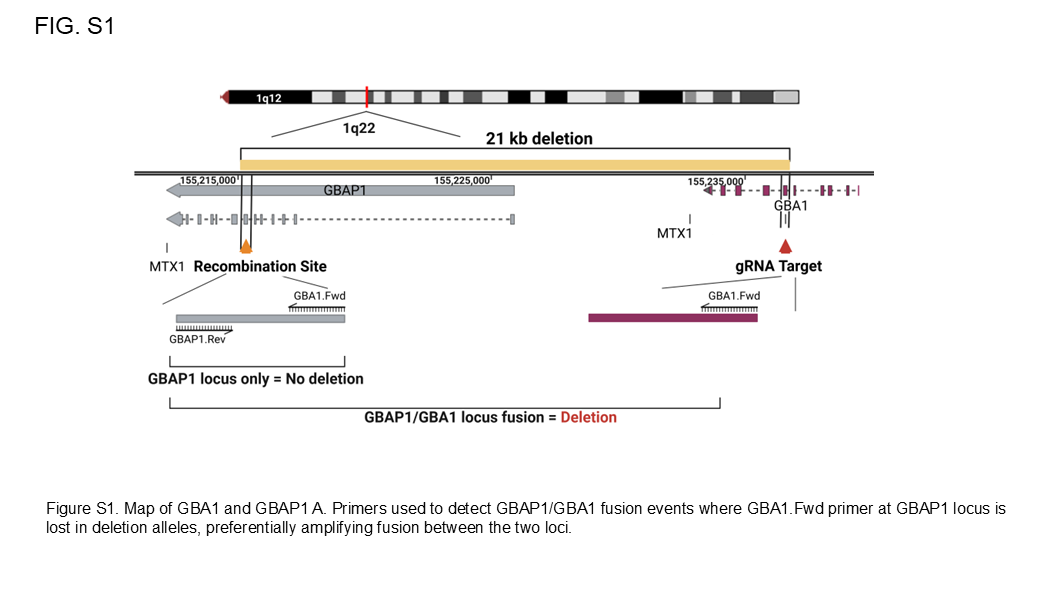

Supplement: Supplementary file 5 [file Image1.tif]
